# Healthcare workers potentially exposed to HIV: an update

**DOI:** 10.1177/01410768221107122

**Published:** 2022-06-20

**Authors:** Kaveh Asanati, Azeem Majeed, Lara Shemtob, Fiona Cresswell

**Affiliations:** 1 Department of Primary Care & Public Health, School of Public Health, Imperial College London, London W6 8RP, UK; 2 Department of Global Health and Infection, Brighton and Sussex Medical School, Brighton, BN1 9PX, UK; 3 MRC-UVRI-London School of Hygiene and Tropical Medicine, Uganda Research Unit, Entebbe, PO Box 49, Uganda

The risk of contracting HIV from an occupational exposure is very low. There have been no reported cases of occupational HIV transmission in the UK since 1999.^1^ From an HIV-positive index case who is not on effective antiretroviral therapy (ART), the risk of HIV acquisition is around 0.3%^2^ (1 in 333) from sharps injuries (e.g. needlestick) and is 0.1%^3^ (1 in 1000 exposures) from splash injuries (e.g. blood splash to eye). Recent published guidelines provide updated information on the use of post-exposure prophylaxis (PEP) following sexual or occupational exposure to HIV.^4^

The number of people living with undiagnosed HIV infection in the UK is at an all-time low. Of all the people living with HIV in the UK (including undiagnosed individuals), over 85% are on effective ART and are virologically suppressed.^4^ There are now compelling data from numerous studies and geographical settings confirming that HIV virological suppression prevents transmission of HIV following sexual exposures (U = U: undetectable equals untransmittable).^5–8^ While there is no evidence about individual level efficacy of ART to prevent transmission following occupational exposures, the same principle is likely to hold true.

With the U = U principle in mind, where the index case has unknown HIV status, the British Association for Sexual Health and HIV (BASHH) recommends the following risk assessment equation to calculate the risk of HIV transmission following a potential exposure^4^:

$$\begin{array}{l} R\mathrm{isk}\;of\;\mathrm{HIV}\;\mathrm{transmission}=\mathrm{risk}\;\mathrm{that}\;\mathrm{index}\;\mathrm{case}\;is\\ \;\mathrm{HIV}-\mathrm{positive}\;\mathrm{and}\;\mathrm{with}\;a\;\mathrm{detectable}\;\mathrm{HIV}\;\\ v\mathrm{iral}\;\mathrm{load}\times \mathrm{risk}\;\mathrm{per}\;{\mathrm{exposure}}^{*}\\ {\;}^{*}1/333\;\mathrm{for}\;\mathrm{needle}\;\mathrm{stick}\;\mathrm{injury}\\ {\;}^{*}1/1000\;\mathrm{for}\;\mathrm{splash}\;\mathrm{injury} \end{array}$$

Data from Public Health England suggest the risk that the index case is HIV-positive with a detectable viral load varies widely in the UK with the highest reported risk in gay and bisexual men in London (32 per 1000) and the lowest risk in non-Black African heterosexual women and men (0.1 and 0.2 per 1000, respectively).^4^ People who inject drugs have an overall risk of being HIV-positive with HIV detectable viraemia of 6.7 per 1000.^4^

In light of these data and using the risk assessment equation, the risk of seroconversion following a potential occupational HIV exposure varies from ∼1/10,500 (0.01%) following a percutaneous injury from a gay or bisexual man in London to ∼1/10,000,000 (0.00001%) following a splash injury from a non-Black African heterosexual woman. Overall, the risk of HIV transmission is very small where the index case has unknown HIV serostatus. For example, the risk of seroconversion from a needlestick injury from a heterosexual British male of unknown status is about 1 in 1,700,000. The risk from a heterosexual British female of unknown HIV status is even less (about 1 in 3,300,000). Therefore, the usual first response measures to a percutaneous injury, including washing the area thoroughly, would suffice as a preventive measure in these cases.

Despite the very low risk of seroconversion, the stress and emotional impact of an occupational exposure to HIV is high.^9–12^ Therefore, counselling on the actual risk of the exposure usually helps addressing stress and anxiety caused by the incident.

## HIV PEP

BASHH recommends PEP following a high-risk injury (sharps or mucosal splash) if the index case is known to be HIV-positive and has not been on ART for more than six months with a confirmed suppressed HIV viral load (<200 copies/mL) within the last six months.^4^ Otherwise, PEP is not generally recommended following other potential occupational HIV exposures unless there is a specific factor that significantly increases the likelihood of transmission (e.g. blood bolus injected or a sharps injury from an injecting drug user in the context of a local HIV outbreak in injecting drug users). Individual risk must be assessed on a case-by-case basis and where there is doubt, an expert opinion should be sought.

The recommended first-line PEP regimen by BASHH is tenofovir disoproxil 245 mg/emtricitabine 200 mg and raltegravir 1200 mg once daily.^4^ Pregnancy and breastfeeding are not contraindications to PEP but women should be counselled appropriately. Raltegavir 400 mg twice daily is preferred as the third agent in pregnancy. In case of treatment failure in the index case, expert advice should be sought.^4^ Where indicated, PEP should be started as soon as possible after exposure, preferably within 24 h, but can be considered up to 72 h.^4^ Drug interactions must be discussed – raltegravir binds to divalent cations such as iron, aluminium, magnesium and calcium and forms a complex at the level of the gut, which results in less raltegravir being absorbed. Therefore, concomitant use of antacids, iron supplements and multivitamins should ideally be avoided while on once-daily raltegravir. Other concomitant medication interactions can be checked via the Liverpool drug interaction checker.

HIV screening of the exposed individual should be performed at baseline, with a follow-up HIV test at 10.5–12 weeks from the time of exposure with a fourth generation laboratory assay.^4^ The recommended PEP regimen is generally well tolerated but those receiving PEP should be warned about adverse drug reactions. Very common side effects (≥1/10) include gastrointestinal effects, sleep disturbance and headache, and more common side effects (≥1/100 to <1/10) include insomnia and abdominal pain.^13^ Side effects are usually mild and transient, though where not tolerated, a change to the treatment regimen should be considered.

Despite the very low risk of seroconversion, occupational HIV exposures are extremely anxiety-inducing. Careful risk communication can help in addressing anxiety. PEP is seldom indicated for occupational exposures if the index case is of unknown HIV status, as the transmission risk is very low. PEP is indicated to reduce the transmission risk following high-risk incidents – exposures where the index case is known to be HIV-positive with a detectable viral load – and is most effective if initiated promptly.

## Declarations

### Guarantor

KA.

### Provenance

Not commissioned; peer-reviewed by Neil Snowise and Kieran Fernando.

### ORCID iDs

Kaveh Asanati https://orcid.org/0000-0002-4455-6102

Azeem Majeed https://orcid.org/0000-0002-2357-9858

Lara Shemtob https://orcid.org/0000-0002-1187-6329
